# Gut microbiota community characteristics and disease-related microorganism pattern in a population of healthy Chinese people

**DOI:** 10.1038/s41598-018-36318-y

**Published:** 2019-02-07

**Authors:** Wen Zhang, Juan Li, Shan Lu, Na Han, Jiaojiao Miao, Tingting Zhang, Yujun Qiang, Yanhua Kong, Hong Wang, Tongxin Gao, Yuqing Liu, Xiuwen Li, Xianhui Peng, Xia Chen, Xiaofei Zhao, Jie Che, Ling Zhang, Xi Chen, Qing Zhang, Ming Hu, Qun Li, Biao Kan

**Affiliations:** 1 State Key Laboratory for Infectious Disease Prevention and Control, National Institute for Communicable Disease Control and Prevention, Chinese Center for Disease Control and Prevention, Beijing, 102206 China; 2 Collaborative Innovation Center for Diagnosis and Treatment of Infectious Diseases, Hangzhou, 310003 China; 3 The 2nd Department of Pulmonary Disease in TCM, The Key Unit of SATCM Pneumonopathy Chronic Cough and Dyspnea, Beijing Key Laboratory of Prevention and Treatment of Allergic Diseases with TCM (No. BZ0321), Department of Pulmonary and Critical Care Medicine, Center of Respiratory Medicine, China-Japan Friendship Hospital; National Clinical Research Center for Respiratory Diseases; No. 2, East Yinghua Road, Chaoyang District, Beijing, China 100029; 4 Nephrology Department, Aviation General Hospital of China Medical University, Beijing, 100012 China; 5 Institute of Animal Science and Veterinary Medicine, Shandong Academy of Agriculture Science, Beijing, China; 6 Zigong Center for Disease Control and Prevention, Zigong, China

## Abstract

China’s population accounts for about 1/5^th^ of the world’s total population. Owing to differences in environment, race, living habits, and other factors, the structure of the intestinal flora of Chinese individuals is expected to have unique features; however, this has not been thoroughly examined. Here, we collected faecal samples from healthy adults living in three cities of China and investigated their gut microbiome using metagenomics and bioinformatics technology. We found that 11 core bacterial genera were present in all of the Chinese faecal samples; moreover, several patient characteristics (age, region, body mass index, physical exercise, smoking habits, and alcoholic drink, and yogurt consumption) were found to have different effects on the gut microbiome of healthy Chinese people. We also examined the distribution patterns of disease-related microorganisms (DRMs), revealing which DRMs can potentially be used as markers for assessment of health risk. We also developed a program called “Guthealthy” for evaluating the health status associated with the microbiome and DRM pattern in the faecal samples. The microbiota data obtained in this study will provide a basis for a healthy gut microbiome composition in the Chinese population.

## Introduction

Gut microbiota disorders have proven to be symptomatic or indicative of a predisposing cause to several diseases, such as allergies, obesity, diabetes, and even mental illness, and appear to affect cancer immunotherapy treatment^1–10^. Population-scale studies on the human microbiome, especially the gut microbiome, including Metagenomics of the Human Intestinal Tract (MetaHit)^11^, the NIH Human Microbiome Project (HMP)^12,13^, the MicroBiome Quality Control (MBQC) project^14^, the National Microbiome Initiative (NMI)^15^, and the American Gut project (AGP) (https://www.indiegogo.com/projects/american-gut/#/), which have aided in understanding the relationship between gut microbiota and health, have been conducted in several countries^13,16,17^.

Owing to differences in environment, race, living habits, and other factors, the structure of the intestinal flora of Chinese individuals is expected to have unique features; however, this has not yet been thoroughly and fully examined. In 2015, Zhang *et al*. performed a large structure survey of fecal microbiota in 314 young adults from 7 ethnic groups living in China^18^. In 2017, Brian *et al*. collected and examined the gut microbiota of 1000 Chinese healthy individuals who spanned ages from 3 to over 100 years^19^. However, the first research was only focusing on young people and the features between different ethnic groups not considering the effect of other living factors. And 7 of 8 age groups in the second study were sampled from 3 cities with a very close distance in one province of China, thus this could not fully represent the baseline microbiota composition for the total Chinese population. Since 2016, the Chinese Center for Disease Control and Prevention (China CDC) has investigated healthy Chinese people and their gut microbiome, including through an epidemiological survey in multiple regions, faecal sampling from healthy people, and an examination of the microbiome using metagenomics and bioinformatics technology. In this paper, a report on the current research results is summarised to clarify the characteristics of intestinal flora in healthy Chinese people and to evaluate the influence of different life factors, as well as the distribution pattern of disease-related microorganisms (DRMs) in healthy Chinese people.

## Results

### Variability of the faecal microbiome in healthy Chinese people

Between 2016–2017, we pre-tested and examined 268 human faecal samples from people living in three cities of China. Detailed information about the inclusion/exclusion criteria is listed in the Methods and Supplemental sections. Compared to the criteria of the HMP in the USA, the criteria in this study was conducted with more stringent standards (Table 1). Several properties shown to be related to human gut microbe composition were surveyed, such as dietary habits and medical history of the family. Moreover, all samples obtained from people with high blood glucose values or high blood pressure were excluded, as well as people with a history of drug use, infusion, and constipation or diarrhoea in the month prior to sampling. Finally, we collected faecal samples from 131 healthy Chinese volunteers for this study.

**Table 1:** Detailed information regarding samples in the Human Microbiome Project (HMP) and data obtained in this study.

	16S Samples from HMP	16S Samples from China	WGS Samples from HMP	WGS Samples from China
Sample Num	310	131	139	11
Location	2 regions of the US	3 cities in China	2 regions of the US	3 cities in China
Age	18~40	22~69	18~40	22~52
Male:Female	281:129	80:51	NA	8:3
Body Mass Index	18~35	16~30	18~35	16~30
Blood pressure	<160/100	<140/90	<160/100	<140/90
Blood sugar after diet	NA	<11.1 mmol/L	NA	<11.1 mmol/L
Drug history	No antibiotics in the past 6 months	No any drug in the past month	No antibiotics in the past 6 months	No any drug in the past month
Disease history	No pulmonary, cardiovascular, gastrointestinal, hepatic or renal functional abnormality; No cancer	No 43 kinds of disease for volunteer and his immediate family	No pulmonary, cardiovascular, gastrointestinal, hepatic or renal functional abnormality; No cancer	No 43 kinds of disease for volunteer and his immediate family
Surgery history	No major surgery in the past 5 years	No any surgery history	No major surgery in the past 5 years	No any surgery history
Disorder	No chronic constipation	No any constipation in the past month	No chronic constipation	No any constipation
	No IBD (mild-moderate-severe); No persistent, infectious gastroenteritis, colitis or gastritis, persistent or chronic diarrhea of unknown etiology	No any diarrhea in the past month	No IBD (mild-moderate-severe); No persistent, infectious gastroenteritis, colitis or gastritis, persistent or chronic diarrhea of unknown etiology	No any diarrhea in the past month
	No Female who is pregnant or lactating	No Female who is pregnant, lactating or in menstrual period	No Female who is pregnant or lactating	No Female who is pregnant, lactating or in menstrual period
Diet and lifestyle habbit survery	NA	Yes	NA	Yes
Sequence Platform	454	Miseq PE300	Illumina PE100	Miseq PE300
Sequence Region	V3–V5	V3–V4	WGS	WGS
Avg Read Length (bp)	386	442	96	202
Read Num	11581	264137	98906340	2715115
Genus Num	82	129	275	99
Bacteroides%	54%	27%	63%	46%
Shannon-Wiener	1.73	2.25	NA	NA
Pielou index	0.39	0.47	NA	NA
Coverage%	NA	NA	44%	24%

The volunteers were selected from three geographical regions (Beijing in the north of China, Jinan in the east of China, and Zigong in the southwest of China). The average age of the healthy volunteers was 35.8 (ranging from 22–69 years of age, standard deviation is 12). The ratio of male to female volunteers was 1.57:1. The average height and weight of the study participants were 165.6 cm and 61.6 kg, respectively. Detailed information for the volunteers is listed in Supplemental Table 1.

**Figure 1:**
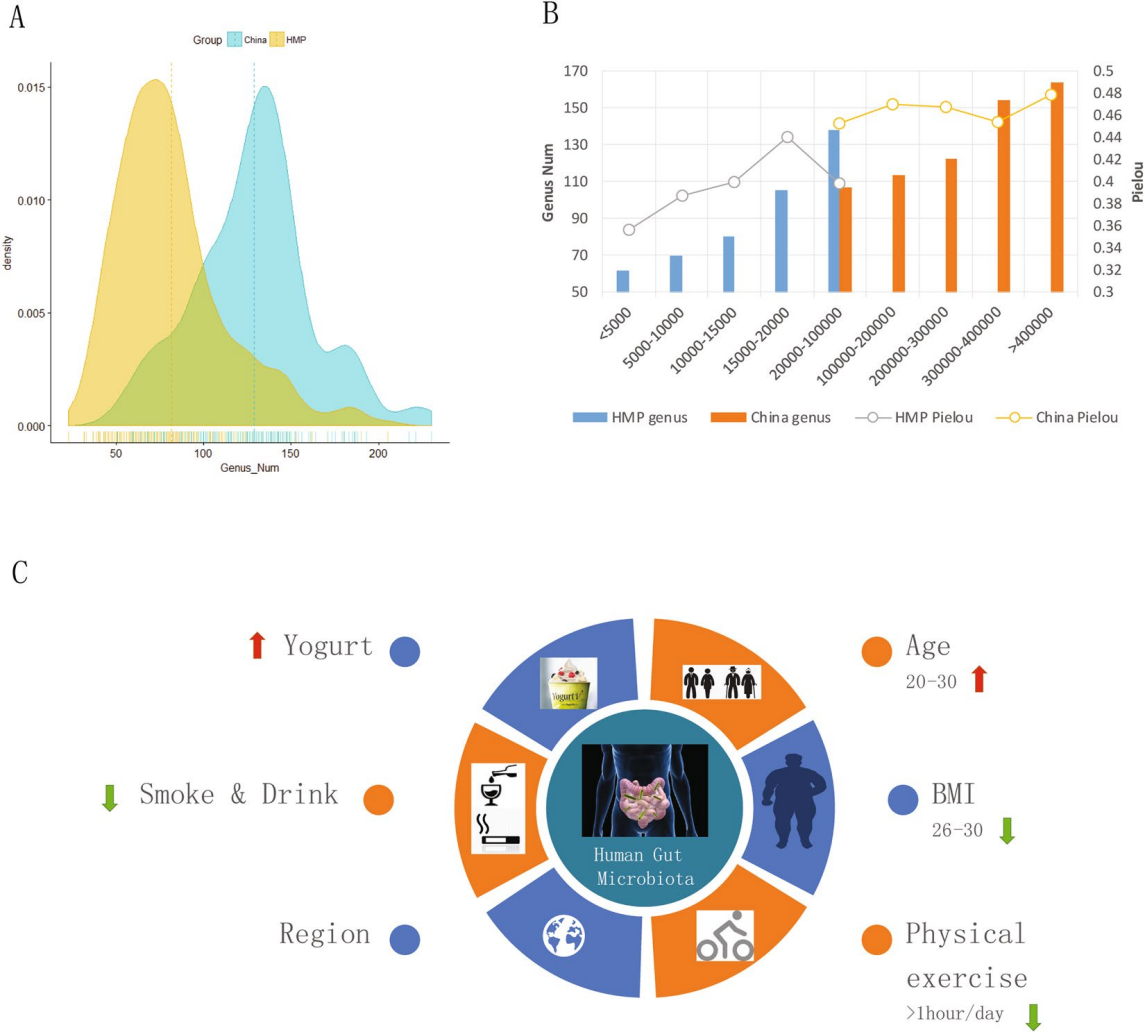
(**A**) Number of bacterial genera identified in the NIH Human Microbiome Project (HMP) and China project. (**B**) The relationship between the identified genus number and read number, as well as Pielou index vs read number in the HMP and our data in this study. (**C**) Factors affecting the gut microbiota. Red arrow represented that the gut microbiota of this group (Age 20–30 or people having yogurt every day) are with genus number and Pielou index values both higher than those in the other group. Green arrow represented that the gut microbiota of this group (BMI 26–30 or people having smoking every day) are with lower genus number and Pielou index values.

Our study revealed that, even in healthy people, the faecal microbiome has high variability, which is also supported by other recent studies^3,4^. In the majority of the samples (49.6%, 65/131), the *Bacteroides* genus comprised the highest proportion of the bacterial population, whereas *Prevotella* was the most abundant genus in 40 samples (30.5%). In the remaining 26 samples, *Faecalibacterium*, *Escherichia*/*Shigella*, *Dialister*, and *Ruminococcus* were the top-ranked genera. Variability was also present in the total number of genera observed. The number of identified genera was 51–230 in 131 of the samples (Fig. 1A and Table 1). The average genus number is 129 (the median is 130; Standard deviation is 33.6).

The variability in the number of identified genera in the faecal microbiome of healthy volunteers was also found in the HMP data (Table 1). However, a higher number of bacterial genera was identified in our samples relative to those found in the HMP. The average genus number was 129, which is significantly higher than that identified in the HMP (Avg: 82; P value = 0.001) (Table 1).

### Common characteristics of faecal samples from healthy Chinese people

Despite the high variability found in these 131 samples, we still identified some common characteristics present among these healthy samples.

First, *Bacteroides* strains were generally present in the samples from healthy Chinese people, with a correspondingly high stable level (Avg: 27%; Standard deviation: 17.9). Based on matching with the 16S database, *Bacteroides* genus was identified in all 131 samples (100%), and *Bacteroides* was the most abundant genus in the 49.6% samples. The high percentage of *Bacteroides* sp. was also supported by the WGS data. The average percentage of *Bacteroides* sp. was 46% (Standard deviation: 22.2) (Table 1).

Second, 11 bacterial genera, *Alistipes, Bacteroides, Blautia, Clostridium, Coprococcus, Escherichia/Shigella, Faecalibacterium, Gemmiger, Parasutterella, Roseburia, and Ruminococcus*, which were identified in all 131 samples, made up the “core genera” found in the healthy Chinese volunteers (Table 2). In contrast to Bacteroides, the other 11 core genera were usually present at low levels in the samples (0.62~12.17%), as shown in Table 2. The WGS results also suggested that *Bacteroides, Blautia, Faecalibacterium, Roseburia, Coprococcus, Ruminococcus*, and *Alistipes* are present in all the project samples from Chinese volunteers. *Clostridium* and *Escherichia/Shigella* were only negative in one sample (ID 1605N03). Due to the lack of reference genome sequences for *Gemmiger*, and *Parasutterella* sp., the presence of the three genera could not be determined in the WGS analysis. *Bacteroides* was also identified in all 311 samples of the HMP project. The 7 of 11 core genus (*Bacteroides, Blautia, Faecalibacterium, Roseburia, Coprococcus, Ruminococcus* and *Clostridium*) identified in this study were also found to be core in the other Chinese project, which covered 314 healthy young adults^18^. Besides 11 bacterial genera, the 16S sequences of Lachnospiracea incertae sedis genus, which belong to the Lachnospiraceae family but the genus could not be sure, was also found to be existing in all 131 Chinese samples and 311 HMP samples. This could be a candidate core genus, but not listed in the core genus in this version for lacking detailed taxonomy information.

**Table 2:** Proportions of the 12 core bacterial genera.

	Avg. (%)	95% Confidence interval(%)
*Alistipes*	1.47	1.20–1.74
*Bacteroides*	27.13	24.05–30.22
*Blautia*	0.62	0.46–0.77
*Clostridium*	5.99	5.25–6.74
*Coprococcus*	0.92	0.70–1.14
*Escherichia_Shigella*	1.36	0.79–1.94
*Faecalibacterium*	12.17	10.61–13.7
*Gemmiger*	1.93	1.51–2.36
*Parasutterella*	0.98	0.71–1.25
*Roseburia*	3.65	3.08–4.22
*Ruminococcus*	3.5	2.55–4.44

Third, the Shannon-Wiener and Pielou evenness index, which represent the community diversity in the gut microbiome of the samples from healthy Chinese people, were 2.17–2.33 and 0.45–0.48 (95% confidence interval), respectively, and all fit the normal distribution.

### Factors that influence the gut microbiota of the Chinese population

Based on the information available for volunteers of the CHMP project, we studied seven candidate factors that influence gut microbiota (BMI, age, region, smoking habits, alcoholic drink and yogurt consumption, physical exercise [PE], and gender). Based on the investigation of genera number, the abundance of the *Bacteroides* genus, and the Shannon-Wiener and Pielou index for gut microbiota, we found that Chinese people with different living factors, such as region, age, yogurt *et al*., have microbiota community divergency (Fig. 1C, Supplemental Figs 1 and 2).

In our project, those volunteers who eat yogurt every day or often eat yogurt (more than 3 times in the past month) were grouped as “Yogurt+ ”, while those who never ate yogurt were classified as “Yogurt−”. A considerable higher number (P value < 0.05) of bacterial genera (Avg: 134 vs 116) as well as a higher Shannon-Wiener (Avg: 2.31 vs 2.00) and Pielou index (Avg: 0.47 vs 0.42) were observed in the Yogurt+ group (Supplemental Fig. 2A), which suggest that yogurt is a candidate factor affecting the human gut microbiota. The relationship between yogurt and the gut microbiota of mice was previously reported in 2011^20^. The Yogurt+ group also had a higher percentage of *Faecalibacterium* and *Bifidobacterium* sp. than that in the Yogurt− group (Supplemental Fig. 3A).

BMI was another factor associated with the gut microbiome community composition. For healthy Chinese people with a higher BMI (26–30), the Shannon diversity indices (Avg: 2.05) were lower than that in people with a lower BMI (BMI 16–20: Avg 2.35 and BMI 21–25: Avg 2.25; P value < 0.05) (Supplemental Fig. 2B). The Pielou evenness index also showed the same tendency, consistent with that in a previous report^21^.

Samples from younger people usually showed a more abundant flora structure in their gut microbiota (Supplemental Fig. 2C). For example, people in Group Age 20–30 have significantly higher Shannon-wiener (2.41) and Pielou index (0.49) than those in Group Age 30–40 (Shannon-wiener:2.09; Pielou:0.44) with P value < 0.05.

People from the three different cities examined in this study also had a different gut community composition, with the Shannon diversity indices and Pielou evenness index showing significant divergence among the groups (Supplemental Fig. 2D). We used Adonis analysis to explore different associations between variation in gut microbiota and host characteristics. By calculating Bray-Curtis distance between samples, we found that Regions exerted the strongest effect (Supplemental Table 4), exceeding the effect of other host factors, which was also supported by the result of He *et al*.^22^.

As is widely known, smoking and alcohol consumption are behaviors associated with health risk. In this study, we also found that these factors influence the human gut microbiota. People who smoke (Avg: 39% vs 24%) or drink alcohol (Avg: 38% vs 27%) had a significantly higher(P < 0.05) proportion of *Bacteroides* than those who did not (Supplemental Fig. 2E,F). Chinese volunteers who smoked one or more times per day also had significantly lower Shannon diversity indices and Pielou evenness index than those volunteers who never smoke (Avg Shannon: 2.07 vs 2.29; Avg Pielou: 0.43 vs 0.47; P value for T test <0.05; Fig. 1C and Supplemental Fig. 2E,F).

Surprisingly, people who spent more time engaging in PE (>1 h/day) had a lower number of bacterial genera as well as Shannon diversity and Pielou index than that in those people without PE habits (Avg Genus Num: 104 vs 142; Avg Shannon: 1.98 vs 2.39; Pielou: 0.43 vs 0.48; P value < 0.05; Supplemental Fig. 2G).

Based on the PCA analysis (Supplemental Fig. 2H), there was no significant difference for genus number, the percentage of *Bacteroides*, and the Shannon-Wiener and Pielou index between the gut microbiota of female and male volunteers. Additionally, no significant divergence was found between people who eat fruit every day and those who never consume fruit (Supplemental Figs 1 and 2I). There was also no evidence supporting that daily sleep time is a factor that influences the human gut microbiota; there was no significant difference between people who sleep more than 8 h per day than those who sleep 4–6 h per day (Supplemental Fig. 2J).

Placing people into different groups based on their living habits also revealed different bacterial genera composition (Supplemental Fig. 3A–J). For example, *Faecalibacterium* was found at a higher percentage in females (Supplemental Fig. 3G) or in people with a lower BMI (Supplemental Fig. 3B).

### DRMs in healthy people

In this study, we investigated the distribution of 182 DRMs in healthy people; these DRMs have unique 16S region sequences at the species level, thus allowing them to be correctly evaluated at the species level in this study.

**Figure 2:**
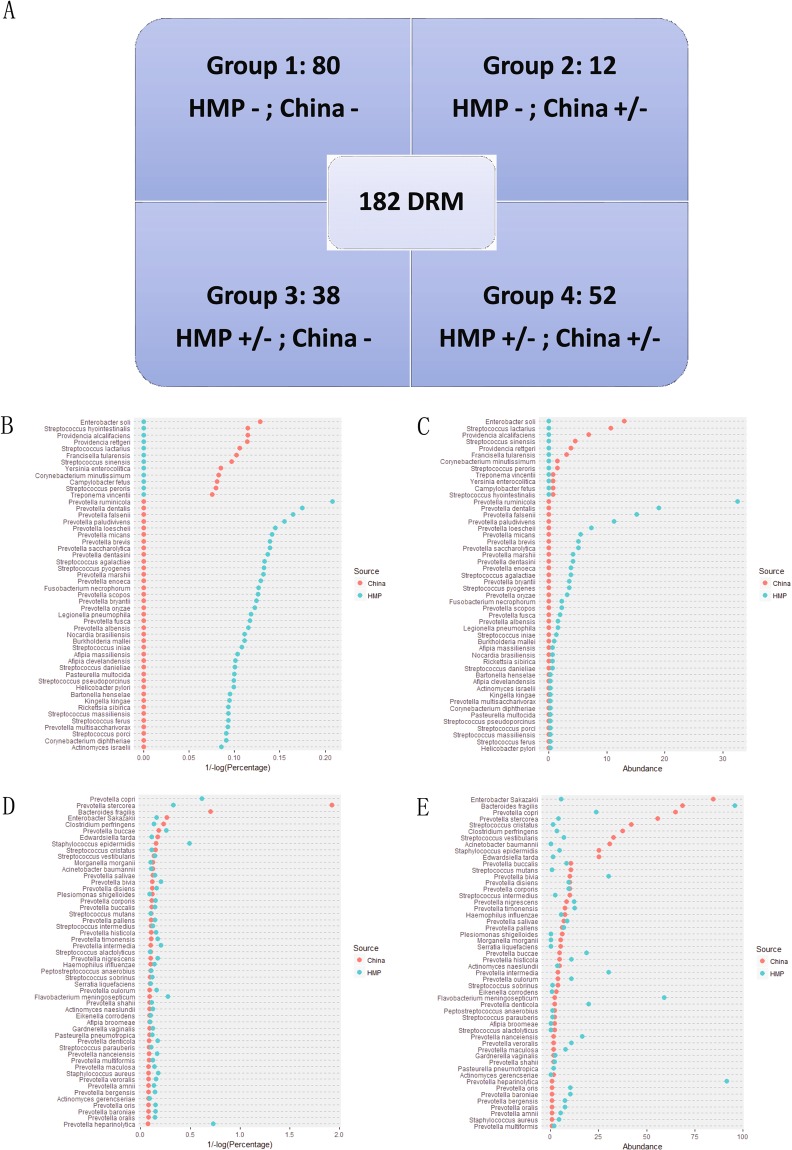
(**A**) Four disease-related microorganism (DRM) groups based on their presence in healthy samples from the HMP and our data in this study. (**B**) Percentage (positive samples/total samples) of Group 2 and Group 3 microorganisms in healthy Chinese people. (**C**) Average abundance level of Group 2 and Group 3 microorganisms in healthy Chinse people. (**D**) Percentage (positive samples/total samples) of Group 4 microorganisms in healthy Chinese people. (**E**) Average abundance level of Group 4 microorganisms in healthy Chinese people.

These 182 DRM species (Supplemental Tables 2 and 3) could be divided into four groups based on their presence in samples from the HMP and China (Fig. 2A and Supplemental Table 3). For group 1, 80 DRMs were not found in any healthy samples, including *Brucella ovis and Chlamydia trachomatis*, which are clearly related to infectious disease and could be seen as a human health risk marker. In other words, if Group 1 DRM species were detected in human faecal samples, those people would have a higher risk level for the infection. Twelve bacterial species (Group 2) and 38 DRM species (Group 3) were only found in China or in the HMP samples, but not in both. This pattern could mainly be caused by differences in long-term diet habits between the two countries. For example, 44.7% of Group 3 bacterial members are *Prevotella* species, which have been shown to be abundant in people who consume more carbohydrates, especially fiber-rich foods^23^. Fifty-two bacterial DRM species were found occasionally in both the HMP and the Chinese volunteer samples (Group 4); these 52 species are typically conditionally pathogenic bacteria and include *Staphylococcus aureus* (4.5% in HMP and 0.7% in China). The proportion of DRMs was also different between the two projects. For example, the positive percentage of *Prevotella heparinolytica* was 91.6% in the HMP project, which is significantly higher than that detected in the China project (0.76%). *Bacteroides fragilis* are commonly found in most healthy samples, but the positive percentage found in the HMP (95.8%) was higher than that in China (68.7%). For those species that could occasionally be found in samples from healthy volunteers, the existence or not of the DRM species belonging to Group 2, 3, or 4 could not be directly used as a marker of poor health, consistent with what was found for Group 1 DRM species. The percentage level of these DRMs needs to be counted and compared to the reference value for those in healthy samples (Fig. 2B).

### Pipeline to evaluate the health status of people

**Figure 3:**
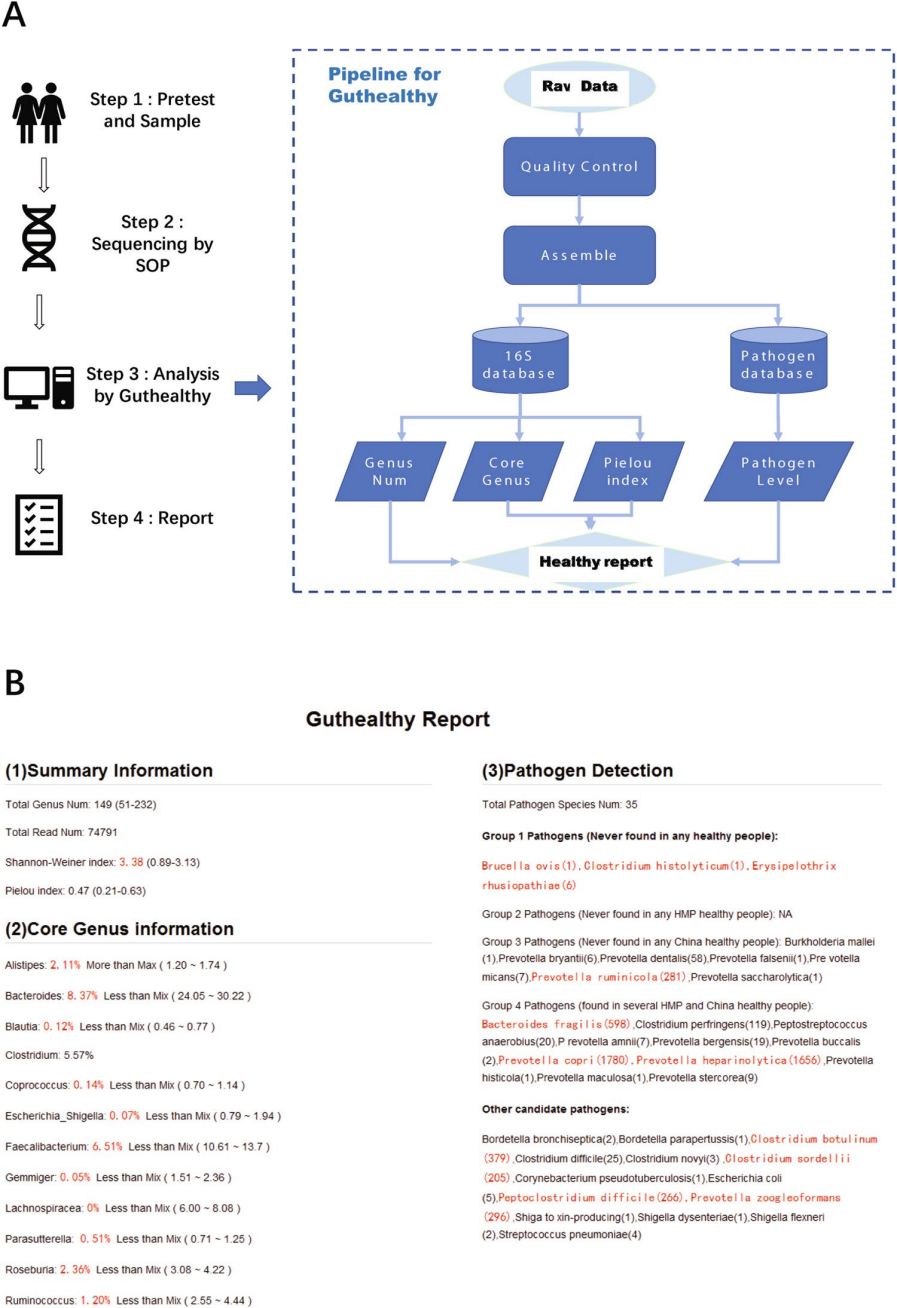
(**A**) Workflow of Guthealthy. (**B**) Example of a Guthealthy test report for a sample from a male Chinese volunteer (201606C05) who took antibiotics for diarrhoea in the previous month. The Guthealthy report for this sample showed several abnormal indexes (shown in red), such as in core genera number and pathogens detected. In this sample, Lachnospiracea sp. was not identified, which are normally present at a high level in all examined samples from healthy Chinese volunteers.

To facilitate comparison with our data, we also developed a program (Guthealthy) for users to quickly obtain information about their candidate health status based on 16S metagenomic results of their gut sample (Fig. 3). The report page is automatically generated and includes genus number, the diversity and evenness index for the microbiome, as well as the existence of core genera and different types of DRMs in the sample. To test the Guthealthy program, we prepared faecal samples from unhealthy people. The criteria for “unhealthy” status included volunteers who had diarrhoea or took antibiotics in the month prior to collection. Figure 3B shows the report for a male Chinese volunteer (201606C05) who took antibiotics for diarrhoea in the previous month. Although this volunteer was without any symptoms of diarrhoea at the time of sampling, his Guthealthy report also showed several abnormal indexes, such as in core genera and pathogens detected. In total, 35 DRM species were identified in his faecal sample. Several *Prevotalla* sp. and *Clostridium* sp. showed more than 200 match reads in the alignments (Fig. 3B). The abnormal gut microbiota of this volunteer may have been caused by the use of antibiotics, which could have killed several normal bacterial species as well as increased the inhibition possibility of other DRMs from the environmental and diet.

Guthealthy is a very fast tool, which can run on a local linux system. The whole analysis process took less than 10 minutes. For those interested in using Guthealthy on their own computer, a Docker Imagination for Guthealthy is available for download (https://github.com/zhangwen2001/Guthealthy.git). The current version of Guthealthy only works on a Linux system. The online Chinese version of Guthealthy was also released (http://analysis.mypathogen.org/), and the online English version will be released in the next few months.

## Discussion

This study, specifically geared towards Chinese people, focused on determining the characteristics of intestinal flora in healthy Chinese people, and we found that there are 12 core bacterial genera in Chinese faecal samples, as well as several factors (age, region, BMI, physical exercise, smoking, and alcoholic drink and yogurt consumption) that affect the gut microbiome of healthy Chinese people. This study also examined the distribution pattern of DRMs within healthy Chinese people, and our investigation showed that among 182 DRM, 80 species (Group 1 DRM) were not found in any samples from healthy Chinese people and could be directly used as a health risk marker when evaluating patient health status from clinical samples via the self-developed program “Guthealthy,” With the help of this information, we have advanced the understanding of what defines a healthy gut for Chinese people.

Nonetheless, “healthy” is not equivalent to the “absence of all diseases.” With the rapid development of next generation-sequencing technologies, increasing amounts of research papers and data have been released regarding the relationship between the gut microbiome and different kinds of diseases, such as inflammatory bowel disease, multiple sclerosis, diabetes (types 1 and 2), allergies, asthma, autism, and cancer^6–10^. In the case of most research examining the relationship between a specific disease and the gut microbiome, researchers usually use “no-disease” people as the control group. For example, people with diabetes may be included in the study group and compared to people without diabetes in the control group. However, people lacking diabetes could potentially have asthma or undergone previous surgical operation, which may also influence the gut microbiome of the volunteer. Other related factors, such as age, gender, BMI, and living habits, are usually not fully released in these papers or are not considered in the survey at all. Thus, data from these “no-disease people” cannot be directly used in other research projects, nor combined as a reference dataset for healthy people. In this study, we first released a reference dataset for healthy Chinese people, with the dataset including not only sequencing data but also the age, gender, BMI, blood pressure, blood glucose levels, and history of drug usage and disease occurrence in the past month, as well as lifestyle (smoking and alcohol consumption). Information about the disease history for the volunteers and their immediate family was also released. All the data from these healthy volunteers can, therefore, be used as the control group for further study of the relationship between the human gut microbiome and disease or other factors, such as air contamination. Research results based on the same control group would allow for greater comparability than those obtained using different control group data, as well as helpful for further understand the impact of different living factor for the people health. In this study, we found that young age and normal weight is associated with high diversity, however physical exercise is associated with lower diversity. The actual effect of physical exercise and the gut community still needs further data combined with our data to make it clear.

“Healthy” is also not equivalent to the complete absence of disease-causing microorganisms. In addition to pathogen infection, so many factors have been shown to affect the human gut microbiome^7^. Our study further proved that BMI, age, region, smoking, alcohol drinking, and PE (Fig. 1C) are all candidate factors that influence the gut microbiota community. On the contrary, even for a people without any disorder symptoms, DRMs can also be detected in healthy volunteers. In sample SRS063324, the percentage of *Staphylococcus* was 53.9%, which is significantly higher than the average level (0.13%), and this volunteer was assumed to have an infection of *Staphylococcus* sp., although he had no symptoms of any disorder at the sample collection time. Moreover, 64 DRMs (12 for Group 2 and 52 for Group 4) could occasionally be found in samples from healthy Chinese people. Our study is still ongoing and more samples are being processed to further support our current results. Moreover, we will consider combining further WGS technology to better detect the existence of these DRMs in the samples from healthy Chinese people.

One important objective for the field is to make the health status measurable and comparable. In this study, we found a higher number of bacterial genera in our samples than those from the HMP^12,13^. The average genus number was 129, which is significantly higher than the 82 detected in the HMP (Table 1). The different living habits for volunteers in the two projects may be responsible for differences between the two projects. For example, *Dehalobacter*, which was commonly found in the HMP project (155 samples, 50%) but not in the Chinese samples, was reported to be related to trichloromethane pollution. Trichloromethane, commonly known as chloroform (CF), is a strong inhibitor of microbial metabolic processes, including organohalide respiration. CF was formerly used as an anesthetic and a precursor chemical in the manufacture of chlorofluorocarbons used as refrigerant gases. Currently, CF exists in 474 of the 1,287 priority polluted sites in the United States^23^. It is possible that this led to positivity for *Dehalobacter* sp., which has been shown to transform CF to dichloromethane^24^. The loss of *Dehalobacter* in the Chinese samples should not be caused by the difference in the sequencing depth based on the rarefaction analysis (Supplemental Fig. 4). However, some divergence between our data and HMP did be caused by the difference in sequencing depth used in the two projects. In the China project, the average read number was 264,137 by Miseq (Median: 246,612, Range: 50,156–12,165,211; Standard deviation: 146,172), which is almost 23 times more than that achieved in the HMP data using a 454-sequencing platform. Given the larger amount of sequencing data available in our samples, more bacterial species that are present at low frequencies could be identified. Figure 1B shows that the identified genus number significantly increases with the read number in the HMP project but not in the China project. Thus, to precisely evaluate and compare the health status of people, a standard operation process (SOP) for sequencing is also necessary. During the progress of this study, we developed a series of SOPs for sample selection, DNA processing, sequencing, and analysis pipeline (Methods section and Supplemental Materials), which have been gradually implemented in different China CDC departments for further study. Our project is still ongoing and more samples are being processed, with more data expected to be released in the next phase of the study.

## Methods

### Sample collection

Between 2016 and 2017, 267 faecal samples were sampled from people living in three cities in China (Beijing, Jinan, and Zigong). These three cities are respectively located in the north, east, and southwest regions of China. All volunteers in the groups were given a pre-test and a detailed pre-questionnaire for their age, career, drug/medical history of both themselves and their immediate families, as well as their living habits (smoking, physical exercise, and fruit and alcoholic drink consumption) (Supplemental Materials 1 and 2) to determine the health level of the volunteers. We also measured the height, weight, blood pressure, and blood glucose levels on site, as well as constructed a detailed inclusion/exclusion criteria to filter out candidate factors affecting the microbiome. Detailed information about the questionnaire process and inclusion/exclusion criteria is provided in Supplemental Materials 1 and 2.

Briefly, only adult volunteers were included in this project. People with any symptoms of diarrhoea, constipation, bloody stools, or cold infection in the past month prior to sample collection were excluded. People who took any drugs, oral or injectable, in the past month were also filtered out. By determining the medical history of volunteers and that of their immediate family, people with any of 43 kinds of diseases were also excluded (Supplemental Material 2). For evaluation of blood pressure and blood glucose levels on site, only volunteers with a blood pressure value of 140 mmHg and 11.1 mmol/l blood glucose levels were included for further experiments.

All faecal samples were collected within 24 h after finishing the pre-test and pre-questionnaire. The feces were collected from a disposable bedpan given to the volunteers and not obtained from a flush toilet, to avoid the complication of collecting toilet water. The study was approved by China CDC, all experiments were performed in accordance with relevant guidelines and regulations, and all people provided informed written consent.

### Data collection, sequencing, and quality control analysis

DNA was extracted from samples within 24 h based on the protocol of the QIAamp Fast DNA Stool Mini Kit (Qiagen, Germany). The V3–V4 region of 16S rDNA was amplified in each sample using the primer 341 F 5′-CCTACGGGNGGCWGCAG-3′ and 805 R 5′-GACTACHVGGGTATCTAATCC-3′. We sequenced these fragments using a Miseq platform with paired-end sequencing of 300 bp. Average read number for 131 samples in this study is 264137 (Median: 246612, Range: 50156–12165211). All low quality (<Q20) bases at the end of the reads were trimmed off. After trimming, only reads with ≥200 bp were kept as high quality reads.

The public faecal microbiome data used in this study were downloaded from theHMP database (http://www.hmpdacc.org/). Samples (311) containing the V3–V5 regions of 16S, which are trimmed off to be V3–V4 regions, were used for the following 16S analyses. Whole Genome Sequencing (WGS) data from 139 healthy people were also downloaded from the HMP database. All these samples were obtained from healthy people based on their release information. All sample analysis performed in this study were done with >3,000 sequencing reads for the 16S samples and 100,000 reads for the WGS samples.

All sequencing data in the project as well as metadata have been released in Microbial Genome Database System (http://data.mypathogen.org, ID = ICDC-20180224-143509), as well as been available in the NCBI SRA database (SAMN09531358 to SAMN09531488).

### Analysis pipeline for sequencing the 16S region

Only samples having >3,000 high quality reads for the HMP data and >50,000 high quality reads for the sample from our study were kept for further analysis. The high quality reads were mapped to the 16S database using Bowtie2^25^ (Version 2.2.6, with parameters -x,-q,-f,-S). For the 16S database used in this study, all sequences with confirmed taxonomic relationships and read lengths ranging from 1200 bp to 2000 bp were selected from public 16S rDNA databases, such as the Ribosomal Database Project (RDP)^26^ and the National Center for Biotechnology Information (NCBI), as well as a database constructed from full-length 16S rDNA fragments extracted from the complete genome sequences of nearly 100 pathogens that were collected, cultured, and sequenced by our unit. After the removal of redundant sequences, the remaining 252,567 reads were used as the 16S reference database, covering 15,217 species and 2,094 genera. Only best match results with a cutoff identity of 97% were retained, and the match reads for each genus were counted. Statistical analysis for Principal Component Analysis (PCA) and LefSe and the corresponding graphical results was based on the local program LEFse^27^ and R packages (ggbiplot and ggpubr).

### Detection of DRMs

DRMs identified in this study represent those bacterial species that have been shown to cause or might potentially cause disease in humans, or other bacterial species in the same genus as those that have been proven to cause disease. Based on the “List of human pathogenic microorganisms” (Enacted by China’s Department of Health in 2006), we listed 339 DRMs in total (Supplemental Table 2), which covered 155 Chinese national legal infectious diseases and several pathogens detected in recent outbreaks as well as several novel bacterial species proven to cause disease.

We obtained the 16S sequences of these 339 DRMs from our 16S database, which was described in detail in the previous section. Only sequences with >1400 bp length were included in the DRM 16S database. This DRM 16S database covering 31,062 sequences of 339 bacterial species was used as the target database for DRM detection in this study. For these 31,062 sequences of 339 DRMs, we compared them one by one using BLAST. Millions of 16S sequence comparisons were conducted among the 339 DRMs; notably, only 182 DRM species had unique 16S region sequences on the species level, and the other 168 species had one or more sequences in common with other bacterial species owing to their identical 16S V3–V4 region. Thus, this has the potential to cause bias in the identification results, and DRMs could not be correctly evaluated at the species level in this study. On summary, only those 16S sequences of 182 DRMs that were proven to be unique on the species level were used in the following analysis for DRM detection. In this study, the detection of 182 DRMs in faecal samples from Chinese people and the HMP project was performed using the Burrows-Wheeler Alignment (BWA) program^28^, applying stringent criteria with a 99% identity cutoff for mapping to the 16S DRM database.

### Analysis pipeline for samples with WGS

Each sample yielded >400,000 high quality reads. The high quality reads obtained from each sample were mapped to the bacterial genome database using BLAST+(parameters: -e value 1e-2)^29^. The bacterial genome database was downloaded from NCBI as well as the Ensemble, ENA, and MPD database, which cover the genomes of 1,527 species and 729 genera. Only matches with more than a 50-bp alignment were retained. For multiple alignments, only the best match result was included. For bacterial species sharing similar gene sequences, such as 16S rDNA and housekeeping genes, even if these strains did not exist in the sample, a random match could potentially occur as a result of this similarity. Thus, the total match region was calculated for each genome and only coverage (coverage % = total match region/genome size) of more than 0.1% was considered as a positive result.

### Ethics approval and consent to participate

Our analysis to 131 fecal samples from Chinese healthy people. Investigators have to obtain informed consent before enrolling participants in this study. The study was approved by China CDC, all experiments were performed in accordance with relevant guidelines and regulations, and all people provided informed written consent.

## Electronic supplementary material

Supplemental Information

Supplemental Table 1

Supplemental Table 2

Supplemental Table 3

Supplemental Table 4
