# Supplementary material for: Gut microbiota community characteristics and disease-related microorganism pattern in a population of healthy Chinese people
Source: Sci Rep. 2019 Feb 7;9:1594. doi: 10.1038/s41598-018-36318-y (PMC6367356; doi:10.1038/s41598-018-36318-y)

Gut microbiota community characteristics and disease-related microorganism pattern in a population of healthy Chinese people

Running title: Gut microbiota of a healthy Chinese population

Wen Zhang[1,2], Juan Li[1,2], Shan Lu[1,2], Na Han[1,2], Jiaojiao Miao[1,2], Tingting Zhang[1,2] , Yujun Qiang[1,2], Yanhua Kong[3], Hong Wang[6], Tongxin Gao[4], Yuqing Liu[5], Xiuwen Li[1,2], Xianhui Peng[1,2], Xia Chen[1,2], Xiaofei Zhao[1,2], Jie Che[1,2], Ling Zhang[6], Xi Chen[6], Qing Zhang[5], Ming Hu[5], Qun Li[6], Biao Kan[1,2*]

Supplemental Material 1 Study Inclusion/Exclusion Criteria

Study Inclusion/Exclusion Criteria

Inclusion Criteria:

In order to be eligible for participation in this study, subjects must meet the following criteria:

- Male or female subjects 18 years of age, but not more than 70 years of age at the time of enrollment.
- Must be able to provide signed and dated informed consent.
- Healthy subjects willing and able to provide stool specimens.

Exclusion Criteria:

Any subject who meets any of the following criteria will be excluded from participation in this study:

- Body Mass Index greater than 30 or less than 16.
- Vital signs outside of acceptable range at Screening Visit, i.e., blood pressure >140/90, blood sugar after diet>11.1mmol/L.
- Use of any drugs within the last month:
- History of cancer, tuberculosis, surgery, or other 40 kinds of disease. Detailed list in the following Table.
- History of cancer, tuberculosis, surgery, or other 40 kinds of disease for his immediate family. Detailed list in the following Table.
- History of infusion therapy in the past month.
- History of constipation, hemorrhoids and blood in the stool in the past month.
- History of rectal exams in the past month.

- History of watery or egg flower like diarrhea in the past month.
- History of cold in the past month.
- Female who is pregnant or lactating or in menstrual period.

Supplemental Material 2 Personal Information Questionnaire

- **Personal Information Questionnaire** ID:
- **Basic Information**

Name		Gendor			
Age		Heigth(cm)		Weigth (kg)	
Blood pressure					
Blood sugar after diet					

- **History of Drug or Disease in the past month**

	Other
Whether or not to take antibiotics in the past month(such as cephalosporins, penicillin, etc.) ? Yes☐ No☐	
Whether or not infusion therapy in the past month? Yes☐ No☐	
Whether there is pain or hemorrhoids or blood in the stool in the past month? Yes☐ No☐	
Is there a rectal examination or treatment in the past month? Yes☐ No☐	
Is there a case of watery or egg like diarrhea in the past month? Yes☐ No☐	
Whether there are more than 3 days in the past month without defecation occurs Yes☐ No☐	
Is there any drugs help defecation in the past month? Yes☐ No☐	
Is there any drugs to treat diarrhea in the past month? Yes☐ No☐	
Is there a surgery in the past month? Yes☐ No☐	
Whether other drug history in the past month? Yes☐ No☐	

- **Diet and lifestyle habbit survey**

Whether or not take lactic acid products (such as yogurt) in the past month	☐Once a day	☐Three more times in the past month	☐One or two times in the past month	☐Never

Eating fruit in the past month	☐eat every day		☐once in two or three days	☐occasionally	☐Never
Smoke	☐more than 2 cigarettes a day		☐once in two or three days	☐occasionally	☐Never
Drinking condition	☐drink every day		☐once in two or three days	☐occasionally	☐Never
Exercise or physical activity in the past month	☐more than 1 hour		☐one or two times in a week	☐occasionally	☐Never
Sleep status in the past month	☐more than 8 hours for one day		☐6-8 hours/day	☐4-6 hours/day	☐<4 hours/day
Diet habits	☐light	☐salty	☐sweet	☐hot and spicy	☐fried food

- **Working and living environment**
- ☐No ☐Noise ☐electromagnetic radiation ☐dust pollution ☐Chemical pollution☐Air pollution ☐Pollution of building decoration ☐Cooking fume pollution ☐Biochemical Reagent ☐Other pollution
- **Medical History**

☐Hypertension	☐Diabetes	☐Cirrhosis	☐History of obesity	☐Helocobacter pylori infection
☐Coronary heart disease	☐Hyperthyroidism	☐Pancreatic disease	☐Peripheral vascular disease	☐Chronic cholecystitis
☐Rheumatic heart disease	☐anemia	☐Acute and chronic nephritis	☐Heart failure	☐Chronic breast disease
☐Congenital heart disease	☐Epilepsy	☐Connective tissue disease	☐Chronic obstructive pulmonary disease	☐Blood lipid abnormality
☐Cardiomyopathy	☐Mental Disease	☐Sexually transmitt	☐Osteoporosis	☐Elevated uric acid

		ed disease		
☐Bronchiectasia	☐Neurosis	☐Cancer	☐Gout	☐History of severe allergies
☐Bronchial asthma	☐History of drug abuse	☐Surgery history	☐Rheumatoid arthritis	☐Hereditary disease
☐Emphysema	☐Acute and chronic hepatitis	☐History of severe trauma	☐Cerebral apoplexy	☐Other disease
☐Peptic ulcer	☐Tuberculosis	☐HPV	☐Asthma	

-
- **Medical history of immediate family**

☐Hypertension	☐Diabetes	☐Cirrhosis	☐History of obesity	☐Helocobacter pylori infection
☐Coronary heart disease	☐Hyperthyroidism	☐Pancreatic disease	☐Peripheral vascular disease	☐Chronic cholecystitis
☐Rheumatic heart disease	☐anemia	☐Acute and chronic nephritis	☐Heart failure	☐Chronic breast disease
☐Congenital heart disease	☐Epilepsy	☐Connective tissue disease	☐Chronic obstructive pulmonary disease	☐Blood lipid abnormality
☐Cardiomyopathy	☐Mental Disease	☐Sexually transmitted disease	☐Osteoporosis	☐Elevated uric acid
☐Bronchiectasia	☐Neurosis	☐Cancer	☐Gout	☐History of severe allergies
☐Bronchial asthma	☐History of drug abuse	☐Surgery history	☐Rheumatoid arthritis	☐Hereditary disease
☐Emphysema	☐Acute and chronic hepatitis	☐History of severe trauma	☐Cerebral apoplexy	☐Other disease
☐Peptic ulcer	☐Tuberculosis	☐HPV	☐Asthma	

Supplemental Figure 1. Determination of genus number, *Bacteroides* percentage, Shannon-Winner index, and Pielou index. The X-axis represents the classification of groups based on the attributes of the volunteer (yogurt consumption, BMI, age, region, smoking habits, alcohol drinking frequency, physical exercise, and gender).

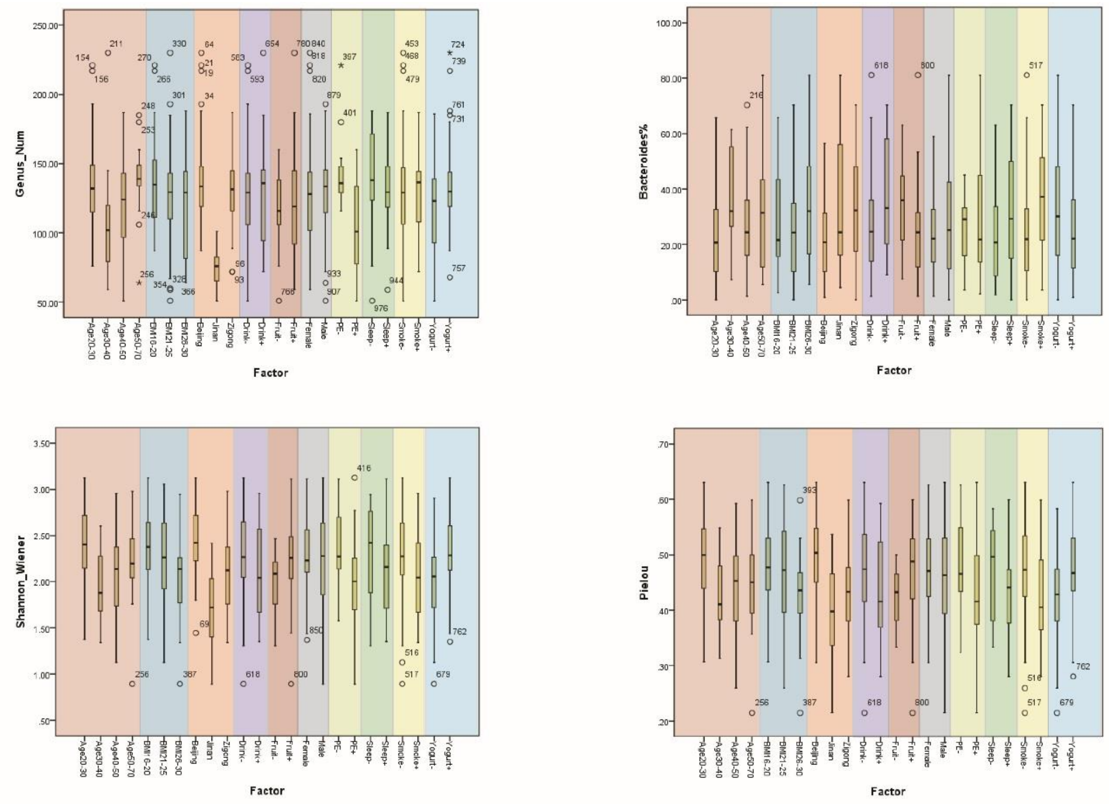

Supplemental Figure 2. PCA of gut microbiota in Chinese people. (A) Yogurt consumption; (B) BMI; (C) age; (D) sample regions/cities; (E) smoking; (F) alcohol drinking; (G) physical experience; (H) gender; (I) fruit consumption; (J) sleep.

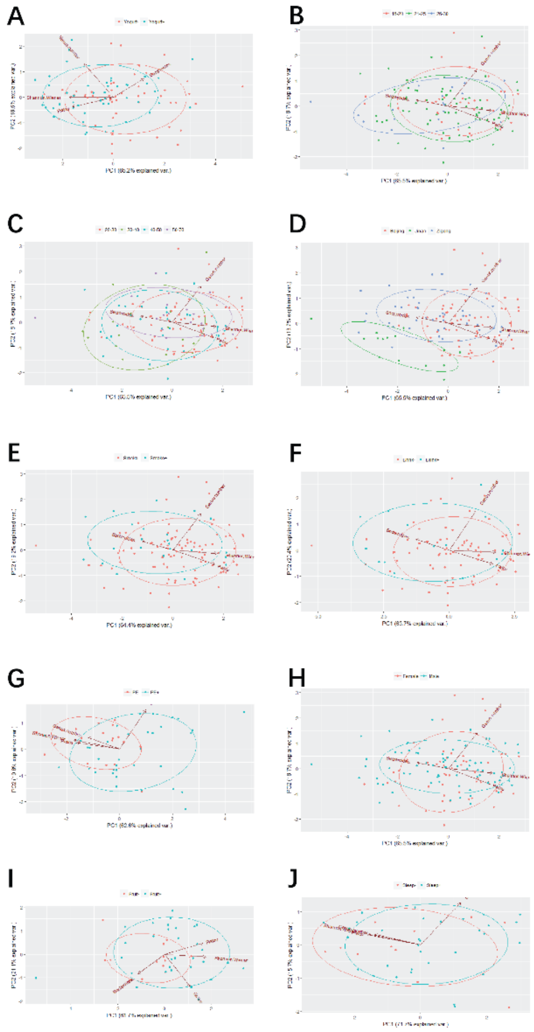

Supplemental Figure 3. LefSe analysis for the gut microbiota in Chinese people. (A) Yogurt consumption; (B) BMI; (C) age; (D) sample regions/cities; (E) smoking; (F) alcohol drinking habits; (G) gender; (I) female vs male population for those with a BMI of 16–20; (J) BMI of 16–25 vs BMI of 26–30 for Chinese females.

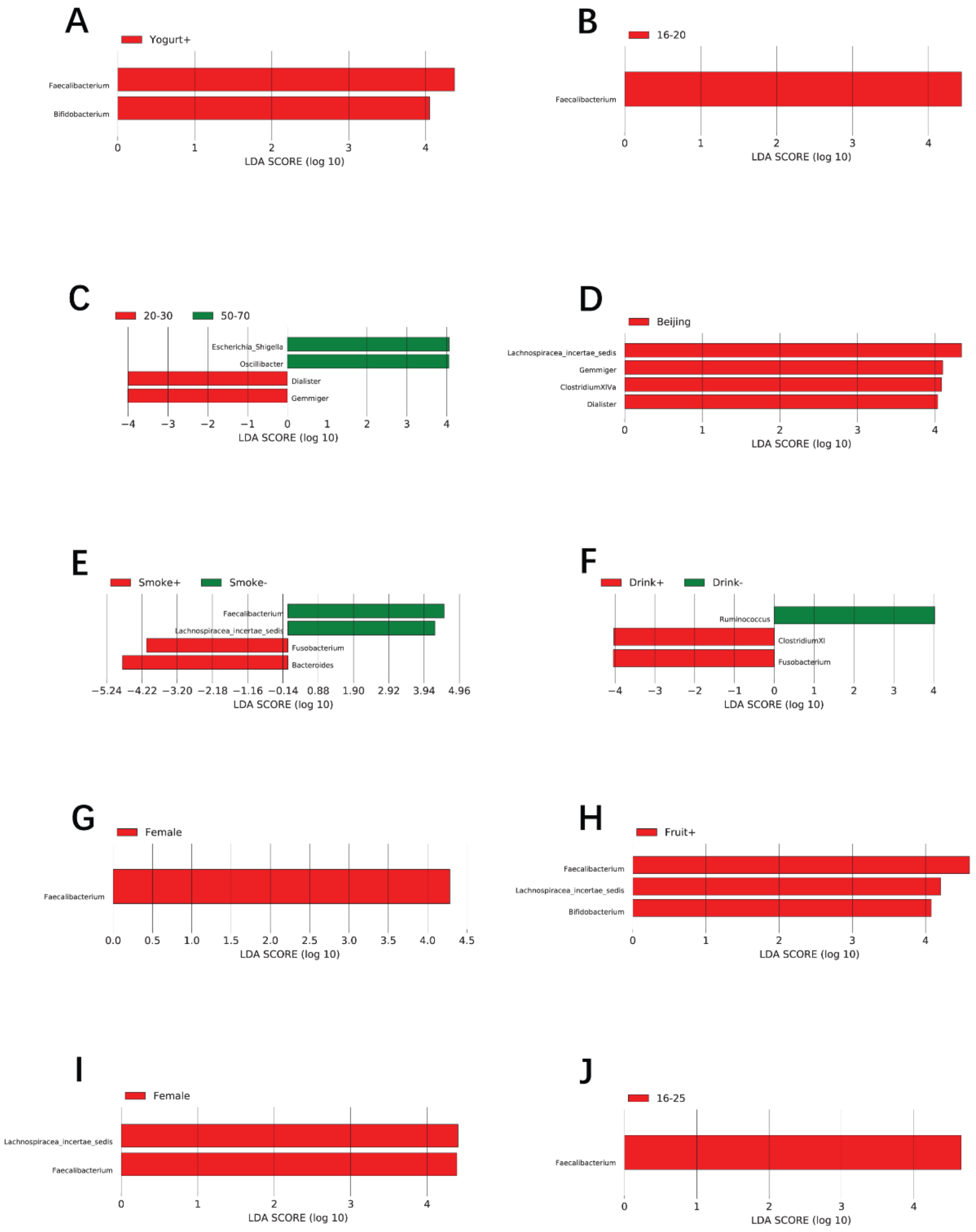
A
Yogurt+
Faecalibacterium
Bifidobacterium
0 1 2 3 4
LDA SCORE (log 10)
B
16-20
Faecalibacterium
0 1 2 3 4
LDA SCORE (log 10)
C
20-30
50-70
Escherichia_Shigella
Oscillibacter
Dialister
Gemmiger
−4 −3 −2 −1 0 1 2 3 4
LDA SCORE (log 10)
D
Beijing
Lachnospiracea_incertae_sedis
Gemmiger
ClostridiumXlVa
Dialister
0 1 2 3 4
LDA SCORE (log 10)
E
Smoke+
Smoke-
Faecalibacterium
Lachnospiracea_incertae_sedis
Fusobacterium
Bacteroides
−5.24 −4.22 −3.20 −2.18 −1.16 −0.14 0.88 1.90 2.92 3.94 4.96
LDA SCORE (log 10)
F
Drink+
Drink-
Ruminococcus
ClostridiumXI
Fusobacterium
−4 −3 −2 −1 0 1 2 3 4
LDA SCORE (log 10)
G
Female
Faecalibacterium
0.0 0.5 1.0 1.5 2.0 2.5 3.0 3.5 4.0 4.5
LDA SCORE (log 10)
H
Fruit+
Faecalibacterium
Lachnospiracea_incertae_sedis
Bifidobacterium
0 1 2 3 4
LDA SCORE (log 10)
I
Female
Lachnospiracea_incertae_sedis
Faecalibacterium
0 1 2 3 4
LDA SCORE (log 10)
J
16-25
Faecalibacterium
0 1 2 3 4
LDA SCORE (log 10)

Supplemental Figure 4. Rarefaction analysis for *Dehalobacter* in HMP and China samples.

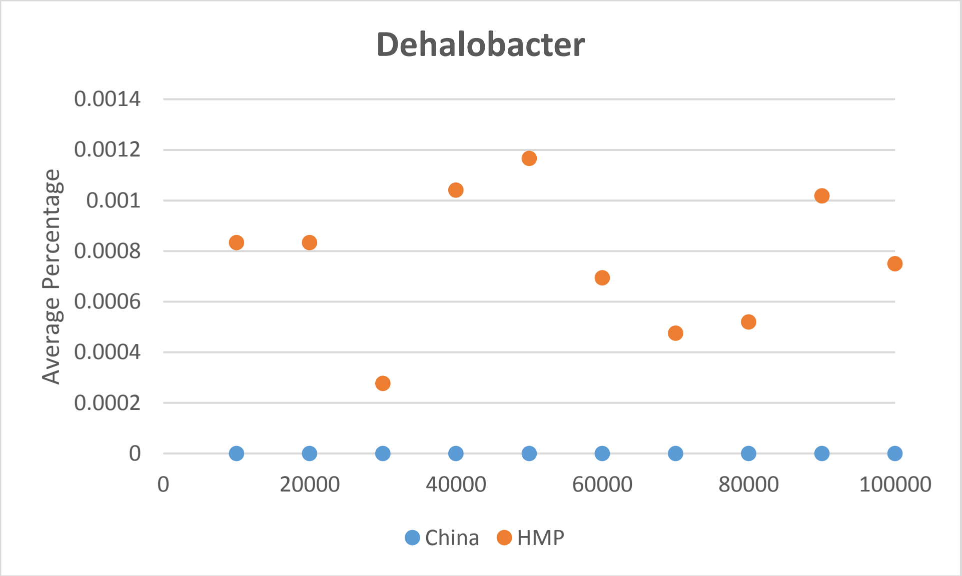

Supplement: Supplementary file 1 — Supplemental Information [file 41598_2018_36318_MOESM1_ESM.pdf]
